# A Novel Mutation of *ATP7B* Gene in a Case of Wilson Disease

**DOI:** 10.3390/medicina57020123

**Published:** 2021-01-29

**Authors:** Cigdem Yuce Kahraman, Ali Islek, Abdulgani Tatar, Özlem Özdemir, Adil Mardinglu, Hasan Turkez

**Affiliations:** 1 Department of Medical Genetics, Faculty of Medicine, Ataturk University, 25240 Erzurum, Turkey; cigdem.kahraman@atauni.edu.tr (C.Y.K.); agtatar@gmail.com (A.T.); 2 Department of Pediatric Gastroenterology, Faculty of Medicine, Ataturk University, 25240 Erzurum, Turkey; ali.islek@atauni.edu.tr; 3 Department of Molecular Biology and Genetics, Faculty of Science, Erzurum Technical University University, 25250 Erzurum, Turkey; ozlem.ozdemir@erzurum.edu.tr; 4 Centre for Host-Microbiome Interactions, Faculty of Dentistry, Oral & Craniofacial Sciences, King’s College London, London SE1 9RT, UK; 5 Science for Life Laboratory, KTH-Royal Institute of Technology, SE-17121 Stockholm, Sweden; 6 Department of Medical Biology, Faculty of Medicine, Ataturk University, 25240 Erzurum, Turkey; hasanturkez@yahoo.com

**Keywords:** novel mutation, *ATP7B*, Wilson disease, rare disorder, copper, liver failure

## Abstract

Wilson disease (WD) (OMIM# 277900) is an autosomal recessive inherited disorder characterized by excess copper (Cu) storage in different human tissues, such as the brain, liver, and the corneas of the eyes. It is a rare disorder that occurs in approximately 1 in 30,000 individuals. The clinical presentations of WD are highly varied, primarily consisting of hepatic and neurological conditions. WD is caused by homozygous or compound heterozygous mutations in the *ATP7B* gene. The diagnosis of the disease is complicated because of its heterogeneous phenotypes. The molecular genetic analysis encourages early diagnosis, treatment, and the opportunity to screen individuals at risk in the family. In this paper, we reported a case with a novel, hotspot-located mutation in WD. We have suggested that this mutation in the *ATP7B* gene might contribute to liver findings, progressing to liver failure with a loss of function effect. Besides this, if patients have liver symptoms in childhood and/or are children of consanguineous parents, WD should be considered during the evaluation of the patients.

## 1. Introduction

Wilson disease (WD) (OMIM# 277900) is an autosomal recessive inherited disorder characterized by excess copper storage in different tissues, including the liver, brain, and the corneas of the eyes. WD is a rare disorder that occurs in approximately 1 in 30,000 individuals. The main symptoms and signs are related to liver disease and/or neurological and psychiatric problems. The age of onset ranges from 6 to 45 years, but WD often occurs in the teenage years. The clinical presentations of WD are highly varied, prominently consisting of hepatic and neurological conditions. The hepatic conditions include acute and chronic liver diseases, as well as fulminant hepatic failure and liver cirrhosis. The neurological conditions consist of extrapyramidal symptoms, such as dystonia and tremor, and the neuropsychiatric symptoms include mood disorder, neurotic behaviors, and disorganization of the personality [1,2]. The most common ophthalmologic finding in patients with WD is Kayser-Fleischer (K-F) rings, which deposit copper in the Descemet’s membrane of the cornea. The rings are present in 90 to 100% of patients with WD [3].

The homozygous or compound heterozygous mutations in the *ATP7B* gene, which encodes the copper-transporting ATPase 2 protein, lead to WD formation. The *ATP7B* gene is located on chromosome 13q14.3 and contains 21 exons [4]. The defective gene causes reduced copper excretion and eventually copper accumulation in the liver, central nervous system (CNS), cornea, joints, kidney, and heart muscle, contributing to WD’s clinical features [2]. The diagnosis of WD is complicated because of its heterogeneous phenotypes. Especially in pediatric patients, the clinical picture may vary significantly, from mild signs of abnormal liver enzymes to acute liver failure requiring liver transplantation. Clinical findings may present with subtle symptoms, such as asymptomatic hepatomegaly, transaminitis, movement disorders, school failure or mood disorders. Thus, the molecular genetic analysis encourages early diagnosis and treatment, and the opportunity to screen individuals at risk [5,6].

Cu chelators, such as D-penicillamine, trientine, and dimercaptosuccinic acid, are effective agents against liver symptoms and damage in patients with WD. Zinc salts are used for both asymptomatic and symptomatic patients. WD patients should be treated individually. Liver transplantation is the only effective treatment when drug therapy does not work, and acute liver failure or severe liver findings occur [5].

## 2. Case

In the present study, we report a case with a novel mutation in WD. A 10-year-old boy, the son of consanguineous parents, was admitted to our hospital due to swelling of the legs and abdomen, jaundice and weakness in the last 10 days. No symptoms were recently described in favor of fever or systemic infection. In the physical examination, icteric scleras, distended abdomen and ascites, and pitting pretibial edema (1+) were positive.

In the laboratory examinations, transaminase elevation, hypoalbuminemia, bicytopenia (anemia and thrombocytopenia), mild hemolysis findings in blood smear, hypouricemia and normal alkaline phosphatase levels were observed (Table 1). Abdominal/doppler ultrasonography revealed cirrhosis in the liver parenchyma and ascites. The ceruloplasmin level was low, and the 24 h urine copper level was high. Serological tests of other viral agents and autoimmune markers were negative. Eye examination revealed bilateral Kayser–Fleischer rings. The patient was diagnosed as WD with these findings.

**Figure 1:**
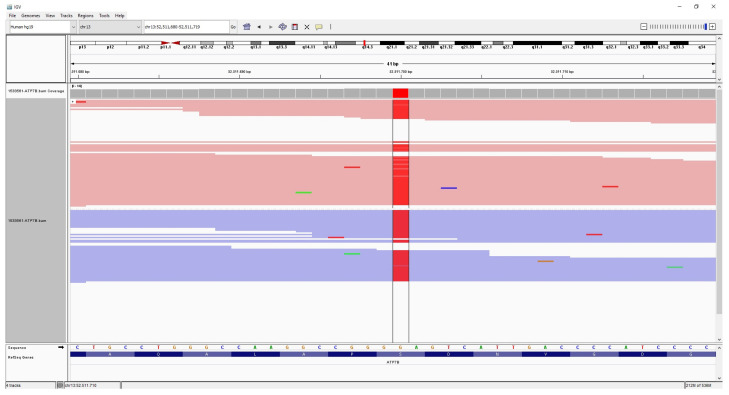
The patient’s integrative genomics viewer (IGV) image of homozygote mutation (red color) c.3815C > A p.S1272Y in *ATP7B*.

**Figure 2:**
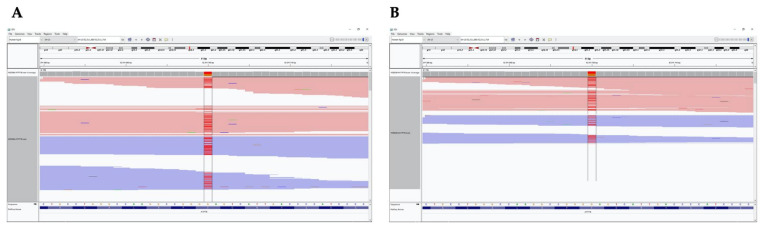
IGV images of heterozygote mutation (red color) c.3815C >A p.S1272Y in *ATP7B* inherited from the patient’s father (**A**) and mother (**B**).

The *ATP7B* gene sequencing was performed with a pre-diagnosis of WD. Whole *ATP7B* gene sequencing was performed on the Illumina MiSeq next-generation sequencing (NGS). The results were evaluated by the Qiagen Clinical Insight (QCI) interpretation software. Sequence analysis of the *ATP7B* gene revealed a novel homozygote missense mutation that was not reported previously in the literature. The mutation was c.3815C > A p.S1272Y within exon 18 of the *ATP7B* (NM_000053.4) gene (Figure 1), and was evaluated as likely pathogenic according to in silico analysis (according to The American College of Medical Genetics and Genomics (ACMG) criteria; PM1, PM2, PP3). The QCI interpretation software indicated that the mutation has a loss of function effect and is located in a mutational hotspot—a critical and well-established functional domain. The parents who were screened for the mutation in question. NGS analysis revealed that both the parents were heterozygote carriers of the mutation (Figure 2).

In the clinical follow up of the patient, the signs of liver failure did not regress under treatment of edema-ascites and combined chelating therapy with metal-captase and zinc. Liver transplantation was performed in the 5th month of follow-up due to decompensated liver failure. 

## 3. Discussion

WD is classically diagnosed with clinical outcomes and laboratory tests. Not all findings are seen together in most patients, and WD manifests as liver dysfunction or decreased ceruloplasmin levels with unknown reasons. However, the diagnosis is difficult if there are mild and nonspecific findings, and may be delayed until more severe clinical outcomes are seen. In such situations, molecular genetic tests are valuable for early diagnosis and treatment. Molecular genetic screening of the patient’s family members reveals carriers, or permits the early detection of other mutant individuals. In addition to the neurological examination, biochemical markers (low ceruloplasmin), Kayser–Fleischer rings, and genetic biomarker (*ATP7B*) are common approaches to WD diagnosis [7,8,9].

WD has an extremely high fatality rate. Early diagnosis and medical therapy are of great importance for WD patients. It is stated in the literature that WD begins in childhood–adolescence, and liver findings are prominent in this age group [1,10]. Inconsistent with the literature, our patient’s findings started at the age of 10, and liver findings were prominent. There were no neurological symptoms.

More than 800 pathogenic mutations of the *ATP7B* gene have been described in WD patients, and most of them are missense mutations. Missense mutations were revealed in 50%, whereas nonsense and frameshift mutations were present in 16.7% of the alleles [11]. Nonsense and frameshift mutations are associated with the earlier and more severe WD manifestation [12]. Compound heterozygosity is the most common genotype in WD cases. Genotypes differ by geographic regions; *R778L* mutations are common in the Asian population and *H1069Q* mutations are common in the European population. The other mutations occur in less than 10% [1].

We found a novel missense mutation in the *ATP7B* gene of our patient via NGS analysis. It was a homozygous mutation within exon 18 (c.3815C > A p.S1272Y). The parents were related, and both were carriers of the mutation. This mutation was not reported in the literature. In silico analysis revealed the mutation to be likely pathogenic. With the clinical findings, we evaluated this mutation as disease-causing. Although the information about the genotype–phenotype correlation is not consistent, it has been reported that ethnicity, modifier genes and protein functionality affect this relationship [1]. Thus, we speculate that the missense mutation, p.S1272Y in the *ATP7B* gene, might contribute to hepatic symptoms that may progress to liver failure due to the loss of function effect of the mutation located in a mutational hotspot, that is, a critical and well-established functional domain.

## 4. Conclusions

We reported a WD case with a novel homozygous mutation that had a loss of function effect. Since the mutation was localized in a hotspot and critical area, it might be responsible for the progression of liver disease. The impact of the mutation on protein function is important in genotype–phenotype correlation [1]. In WD, molecular genetic analysis is important for early diagnosis and treatment, and for identifying family members at risk. The symptoms of WD range from mild to severe symptoms. Differential diagnosis of WD should be made in the presence of benign hepatic findings or, to a lesser extent, neurological findings, and excluded with molecular genetic tests if necessary. Besides this, if hepatic findings are observed in consanguineous parents’ children, WD should be carefully considered during the evaluation of the patients.

## Figures and Tables

**Table 1:** Patient’s laboratory findings.

Parameters			
Hemoglobin (g/dL)	11.1	INR	5.11
WBC count (m^3^)	11,900	Hepatitis A and E IgM	Negative
Thrombocyte count (m^3^)	113,000	Hbs ag	Negative
ALT (U/L) (5–35)	178	HBc IgM	Negative
AST (U/L) (5–35)	570	Hbs ab	Negative
GGT (U/L) (3–22)	192	HCV ab	Negative
ALP (U/L) (42–362)	190	Parvovirus IgM and IgG	7.5 (negative)
LDH (U/L) (110–295)	418	EBV IgM	Negative
Albumin (g/dL) (3.5–5.2)	2.11	EBV IgG	Negative
Uric acid (mg/dL)	1	Ceruloplasmine (mg/L) > 200)	167
Total bilirubin (mg/dL) (0.3–1)	5.3	24 h urine copper (mcg/d) (<40)	1286
Direct bilirubin (mg/dL) (<1)	2.6	Autoimmune antibodies (ANA, ASMA, anti-LKM)	Negative
PT (min)	61.1	Immunoglobulin G (g/L) (6.08–15.72)	39.9

WBC, white blood cell count; ALT, alanine aminotransferase; AST, aspartate aminotransferase; GGT, gamma-glutamyl transferase; ALP, alkaline phosphatase; LDH, lactate dehydrogenase; PT, prothrombin time; INR, international normalized ratio; IgM, immunoglobulin M; IgG, immunoglobulin G; HBc, hepatitis core; ab, antibody; Hbs ag, hepatitis B surface antigen; HCV, Hepatitis C; EBV, Epstein Barr Virus; ANA, anti-nuclear antibody; LKM, liver kidney microsomal antibody; ASMA, anti-smooth muscle antibody.

## Data Availability

Data is contained within the article.
